# Workplace and non-workplace cannabis use and the risk of workplace injury: Findings from a longitudinal study of Canadian workers

**DOI:** 10.17269/s41997-023-00795-0

**Published:** 2023-07-31

**Authors:** Nancy Carnide, Victoria Landsman, Hyunmi Lee, Michael R. Frone, Andrea D. Furlan, Peter M. Smith

**Affiliations:** 1 Institute for Work & Health, Toronto, Ontario Canada; 2 Dalla Lana School of Public Health, University of Toronto, Toronto, Ontario Canada; 3 Department of Psychology, University at Buffalo, The State University of New York, Buffalo, NY USA; 4 Toronto Rehabilitation Institute, University Health Network, Toronto, Ontario Canada; 5 Department of Medicine, Temerty Faculty of Medicine, University of Toronto, Toronto, Ontario Canada; 6 Department of Epidemiology and Preventive Medicine, Monash University, Melbourne, Victoria Australia

**Keywords:** Cannabis, Occupational injuries, Accidents, occupational, Workplace, Occupational groups, Longitudinal studies, Humans, Cannabis, lésions professionnelles, accidents du travail, lieu de travail, groupes professionnels, études longitudinales, humains

## Abstract

**Objectives:**

Findings of previous studies examining the relationship between cannabis use and workplace injury have been conflicting, likely due to methodological shortcomings, including cross-sectional designs and exposure measures that lack consideration for timing of use. The objective was to estimate the association between workplace cannabis use (before and/or at work) and non-workplace use and the risk of workplace injury.

**Methods:**

Canadian workers participating in a yearly longitudinal study (from 2018 to 2020) with at least two adjacent years of survey data comprised the analytic sample (*n* = 2745). The exposure was past-year workplace cannabis use (no past-year use, non-workplace use, workplace use). The outcome was past-year workplace injury (yes/no). Absolute risks and relative risks (RR) with 95% confidence intervals (CIs) were estimated between workplace and non-workplace cannabis use at one time point and workplace injury at the following time point. Models were adjusted for personal and work variables and were also stratified by whether respondents’ jobs were safety-sensitive.

**Results:**

Compared to no past-year cannabis use, there was no difference in workplace injury risk for non-workplace cannabis use (RR 1.09, 95%CI 0.83–1.44). However, workplace use was associated with an almost two-fold increased risk of experiencing a workplace injury (RR 1.97, 95%CI 1.32–2.93). Findings were similar for workers in safety-sensitive and non-safety-sensitive work.

**Conclusion:**

It is important to distinguish between non-workplace and workplace use when considering workplace safety impacts of cannabis use. Findings have implications for workplace cannabis use policies and substantiate the need for worker education on the risks of workplace cannabis use.

**Supplementary information:**

The online version contains supplementary material available at 10.17269/s41997-023-00795-0.

## Résumé

**Objectifs:**

Les résultats d’études antérieures portant sur la relation entre la consommation de cannabis et les accidents du travail sont contradictoires, probablement en raison de lacunes méthodologiques, notamment les études transversales et les mesures de l’exposition qui ne tiennent pas compte du moment de la consommation. L’objectif était d’estimer l’association entre la consommation de cannabis sur le lieu de travail (avant et/ou pendant le travail) et la consommation en dehors du lieu de travail et le risque d’accident du travail.

**Méthodes:**

Les travailleurs canadiens participant à une étude longitudinale annuelle (de 2018 à 2020) avec au moins deux années adjacentes de données d’enquête constituaient l’échantillon analytique (n = 2 745). L’exposition était la consommation de cannabis au travail au cours de l’année écoulée (pas de consommation au cours de l’année écoulée, consommation en dehors du lieu de travail, consommation sur le lieu de travail). Le résultat était l’accident du travail de l’année écoulée (oui/non). Les risques absolus et les risques relatifs (RR) avec des intervalles de confiance (IC) de 95% ont été estimés entre la consommation de cannabis sur le lieu de travail et en dehors du lieu de travail à un moment donné et l’accident du travail au moment suivant. Les modèles ont été ajustés pour tenir compte des variables personnelles et professionnelles et ont également été stratifiés selon que les emplois des répondants étaient ou non sensibles à la sécurité.

**Résultats:**

Par rapport à l’absence de consommation de cannabis au cours de l’année écoulée, il n’y avait pas de différence dans le risque d’accident du travail en cas de consommation de cannabis en dehors du lieu de travail (RR 1,09, IC à 95% 0,83–1,44). Cependant, la consommation sur le lieu de travail était associée à un risque presque deux fois plus élevé de subir un accident du travail (RR 1,97, IC à 95% 1,32–2,93). Les résultats étaient similaires pour les travailleurs exerçant des activités sensibles à la sécurité et pour ceux qui ne le sont pas.

**Conclusion:**

Il est important de distinguer entre la consommation en dehors du lieu de travail et la consommation sur le lieu de travail lorsqu’on étudie les effets de la consommation de cannabis sur la sécurité sur le lieu de travail. Les résultats ont des implications pour les politiques relatives à la consommation de cannabis sur le lieu de travail et justifient la nécessité d’informer les travailleurs sur les risques liés à la consommation de cannabis sur le lieu de travail.

## Introduction

The legal status of cannabis use continues to evolve worldwide. In Canada, a legal medical cannabis program has existed since 2001, while cannabis for non-medical purposes became legal in October 2018. In the United States, an increasing number of states have implemented regulations to allow medical and non-medical use of cannabis. Alongside these changes have come greater accessibility to cannabis (Myran et al., 2022) and increased use among the working-age population (Fischer et al., 2021; Frone, 2019). Public perceptions of cannabis use have also become increasingly positive (Carliner et al., 2017).

Against this backdrop have been calls for more research into understanding the potential impacts of cannabis use on workplace safety (Howard & Osborne, 2020). The acute cognitive and psychomotor impairments that result from cannabis use (Broyd et al., 2016) have the potential to adversely impact a worker’s ability to perform their work safely, particularly for workers in safety-sensitive occupations (i.e., where impaired performance could result in injury to employees or others and/or damage to the property or environment (Els & Straube, 2016)). Yet, studies examining the relationship between cannabis use and risk of workplace injury have yielded conflicting findings, with some demonstrating a greater risk of workplace injury associated with cannabis use and others finding no association (Biasutti et al., 2020).

The inconsistency in prior findings may have resulted from several critical methodological limitations. First, prior studies have relied primarily on cross-sectional data collection. The lack of temporality between the exposure and outcome is problematic given cannabis may be used therapeutically following injury to treat symptoms, such as pain, sleep problems, and poor mental health (Leung et al., 2022).

Second, consistent with other research on worker substance use (Frone, 2019), most studies on cannabis and workplace injury used context-free measures of self-reported lifetime or past-year use that could include use outside work hours. Frone developed a comprehensive model on the associations of worker substance use to workplace outcomes (injuries, performance, attendance) (Frone, 2019). A key feature of this model is that the temporal context of substance use and impairment is matched to specific work outcomes (two types of attendance outcomes and several performance outcomes, including workplace injuries). Notably, the model suggests that workplace cannabis use (use in close proximity to work) is a more critical risk factor for workplace injuries than non-workplace cannabis use (use outside of work). However, to our knowledge, prior studies on cannabis use and workplace injuries have not considered this distinction. With no widely accepted measure of cannabis-related impairment available, workplace use may act as a proxy for workplace impairment (Frone, 2019).

Finally, most studies of cannabis use and workplace injury have included workers in various occupations and industries without exploring whether underlying job hazards modify this association (Biasutti et al., 2020). As noted in Frone’s model, associations between employee substance use and workplace outcomes may be conditional on several moderating variables, including occupation (Frone, 2019). Combining workers in safety-sensitive and non-safety-sensitive occupations may have attenuated the association between cannabis use and workplace injury, as workers in non-safety-sensitive jobs are inherently less likely to experience an injury due to the nature of their work.

The present study aimed to address these limitations using data from a longitudinal study of Canadian workers. Our objectives were to (1) estimate the association between workplace and non-workplace cannabis use and the risk of workplace injury, compared to no cannabis use; and (2) examine whether the relationship between workplace and non-workplace cannabis use and workplace injury is modified by type of occupation (safety-sensitive versus non-safety-sensitive).

## Methods

### Study design and sample recruitment

Data come from a national, split-panel longitudinal study of Canadian workers (Carnide et al., 2021, 2022). Individuals were eligible to enter the cohort at each study wave if they were at least 18 years of age, currently employed, and working 15 or more hours per week for another person or business employing five or more persons. Respondents were recruited mainly from pre-existing panels of households who agreed to participate in occasional surveys, with a small number also recruited through random digit dialing. At each time point, consenting respondents were recontacted in subsequent waves. Additional replenishment samples of respondents were added at each follow-up wave.

The current analysis is limited to workers participating in at least two adjacent surveys from the first three yearly waves (2018–2020; denoted as Time 1[T1]-Time 3[T3]). The analytic sample included 2745 participants: 445 who only completed the T1 and T2 surveys; 1130 who only completed the T2 and T3 surveys; and 585 who completed all three surveys. This latter group appears twice in the analytic sample, contributing one set of data from T1 and T2 and another set from T2 and T3 (see Supplemental File 1 for more detail on participation and the derivation of the analytic sample).

All respondents provided informed consent to participate. The study protocol was approved by the University of Toronto Health Sciences Research Ethics Board (reference 36019 and 37602).

### Measures

Participants completed surveys online or by telephone. Cannabis use and covariates were assessed at T1 and T2, and workplace injury was assessed at T2 and T3, respectively. Thus, information on cannabis use and covariates was collected a year before the assessment of workplace injury.

#### Outcome: workplace injury

Participants were asked a single yes/no item: “*During the past 12 months, have you experienced an incident that resulted in injury to yourself while working?*”.

#### Exposure: workplace and non-workplace cannabis use

Using questions adapted from general population surveys (Health Canada, 2017; Statistics Canada 2017a), participants were asked about lifetime cannabis use and their frequency of past-year use, ranging from never to 5–7 days per week. Respondents reporting past-year use reported their frequency of using cannabis within 2 h before work, during work (excluding breaks), and during breaks, adapting questions from previous research (Frone, 2006). Respondents were then categorized into one of three groups: no past-year use; past-year non-workplace use (use in the past year, but not before/at work); and past-year workplace use (use in the past year, including before/at work).

#### Cannabis use characteristics

In addition to frequency of cannabis use, information on the self-reported purpose of use and primary method of consumption was also collected.

#### Covariates

##### Personal characteristics 

Data were collected on sociodemographic factors, including age, sex, province/territory, and highest level of education. Measures of self-rated general health, past-year alcohol use frequency, and current frequency of cigarette smoking used items from the Canadian Community Health Survey (Statistics Canada, 2016a).

##### Work-related characteristics 

Using questions from the Canadian Labour Force Survey (Statistics Canada, 2016b), data were collected on average weekly work hours, usual work schedule, job permanency, and job tenure. Workers also reported whether they had a supervisory role in their workplace.

Informed by the OHS Vulnerability Measure (Smith et al., 2015), a new item was developed, asking workers whether they participated in hazardous or safety-sensitive work tasks at least weekly in the past year (e.g., driving a motor vehicle; working from heights 2 m/6.5 feet or more above ground; operating or working close to equipment/machinery/tools).

Respondents were asked about their usual contact with their supervisor in the past year (“*I have a lot of contact with my supervisor during a typical workday*”), with responses ranging from strongly agree to strongly disagree (Frone & Trinidad, 2012). Frequency of performing job duties in front of others was assessed using an adapted item (“*How often do you usually perform your job duties in front of or near other people*”), with responses ranging from never to very often (Frone, 2003).

##### Workplace characteristics 

Industry, workplace size, and workplace smoking restrictions were measured using items from Statistics Canada surveys (Statistics Canada, 2016a, 2016b, 2017b). Finally, a single yes/no item was used to identify workers’ awareness of a formal substance use policy in their workplace.

### Analysis

Descriptive and regression analyses were generated using SAS software version 9.4 (SAS Institute Inc., Cary, NC, USA) and R software (2020).

The relative risks (RR) and associated 95% confidence intervals (CIs) of experiencing a workplace injury associated with workplace and non-workplace cannabis use were obtained from absolute risks (ARs) (Localio et al., 2007), estimated from logistic regression models using the method of predictive margins (or marginal standardization) (Graubard & Korn, 1999). Standard errors were adjusted for clustering to account for the inclusion of 585 respondents who participated in all three surveys and contributed two observations to the analyses. Models compared workplace and non-workplace use with no past-year use. Unadjusted models were initially estimated, followed by a model fully adjusted for personal, work and workplace characteristics (including safety-sensitive work), previous work injury, an indicator for time (T1/T2, T2/T3), and survey mode (online only *n* = 1661, telephone only *n* = 672, mixed *n* = 412). Age, frequency of cigarette smoking, alcohol consumption, work hours, job tenure, and workplace size were treated as continuous, with all other variables treated as nominal. Models were also run separately by safety-sensitive work status.

Respondents lost to follow-up were more likely to be younger, be female, report workplace cannabis use, have less than a post-secondary education, be in a supervisory role, and be in non-permanent jobs. There was also variation across industries (details upon request). To address unit nonresponse, each respondent was assigned a nonresponse adjustment weight, proportional to the inverse of the propensity to participate in the corresponding wave. Weights were applied to the regression analyses and results from unweighted and weighted models were similar (details upon request). Only the weighted regression results are reported.

A total of 123 respondents (4.5%) were missing data, on the injury outcome (*n* = 8), workplace cannabis use (*n* = 42), and/or one or more covariates (*n* = 84) (see Supplementary File 2, Tables S1 and S2). To address item nonresponse, multiple imputation was implemented using a fully conditional specification approach with IVEware version 0.3 (2021). All variables included in the analysis were included in the imputation models, which used 20 imputation cycles. Parameters were estimated in each imputed dataset and pooled using Rubin’s rules (Rubin, 1987).

## Results

### Sample characteristics

Information on the personal, work and workplace, and cannabis use characteristics (using unweighted data) are shown in Supplementary File 2 Tables S1–S4. The mean age of the sample was 46.2, with over half (58.5%) being male. Over a third were in a safety-sensitive job.

### Cannabis and workplace injury in the sample

Among all workers, 65.5% did not use cannabis in the past year, 27.4% reported non-workplace use in the past year, and 7.0% reported workplace use (Table 1, using weighted data). The percentages were similar for respondents in safety-sensitive and non-safety-sensitive jobs.

**Table 1:** Cannabis use status and workplace injury among survey respondents, overall and stratified by safety-sensitive work (weighted data)

	%	95% LCL^a^	95% UCL
Cannabis use			
All respondents			
No past-year use	65.5	63.3	67.7
Past-year non-workplace use	27.4		
Past-year workplace use	7.0	5.8	8.3
Respondents in safety-sensitive jobs			
No past-year use	64.7	61.0	68.5
Past-year non-workplace use	27.8	24.2	31.3
Past-year workplace use	7.5	5.4	9.6
Respondents in non-safety-sensitive jobs			
No past-year use	66.0	63.3	68.7
Past-year non-workplace use	27.3	24.8	29.8
Past-year workplace use	6.7	5.2	8.3
Workplace injury			
All respondents			
Yes	11.3	9.8	12.8
No	88.7	87.2	90.2
Respondents in safety-sensitive jobs			
Yes	22.0	18.6	25.3
No	78.0	74.7	81.4
Respondents in non-safety-sensitive jobs			
Yes	4.9	3.7	6.1
No	95.1	93.9	96.3

Abbreviations: *LCL*, lower confidence limit; *UCL*, upper confidence limit

^a^Confidence intervals are not provided when no data are imputed for a particular category

Overall, 11.3% of workers in the sample experienced a workplace injury. When stratified by safety-sensitive work, 22.0% of workers in safety-sensitive jobs and 4.9% in non-safety-sensitive jobs had a workplace injury.

### Relationship between workplace and non-workplace cannabis use and workplace injury

Table 2 provides the ARs and RRs for the relationship between workplace and non-workplace cannabis use and workplace injury. Among all respondents, the adjusted AR of workplace injury was 10.22% (95%CI 8.45–11.98) for no past-year cannabis use, 11.14% (95%CI 8.68–13.61) for non-workplace use, and 20.13% (95%CI 12.99–27.27) for past-year workplace use. Compared to no past-year use, the risk of experiencing a workplace injury was 1.97 times (95%CI 1.32–2.93) higher among workers reporting workplace use. No statistically elevated association was seen for non-workplace use (RR 1.09, 95%CI 0.83–1.44).

**Table 2:** Absolute risks and relative risks of workplace injury by cannabis use status, among all respondents and stratified by safety-sensitive work (weighted data)

Cannabis use	Absolute risks						Relative risks					
	Unadjusted			Fully adjusted^a^			Unadjusted			Fully adjusted^a^		
	AR	95% LCL	95% UCL	AR	95% LCL	95% UCL	RR	95% LCL	95% UCL	RR	95% LCL	95% UCL
All respondents												
No past-year use	9.74	8.04	11.45	10.22	8.45	11.98	1.00			1.00		
Past-year non-workplace use	11.69	8.19	15.19	11.14	8.68	13.61	1.20	0.86	1.68	1.09	0.83	1.44
Past-year workplace use	24.25	15.23	33.28	20.13	12.99	27.27	2.49	1.65	3.75	1.97	1.32	2.93
Stratified by safety-sensitive work												
Safety-sensitive jobs												
No past-year use	19.25	15.58	22.92	20.14	16.22	24.06	1.00			1.00		
Past-year non-workplace use	23.86	16.12	31.61	23.30	17.80	28.80	1.24	0.86	1.79	1.16	0.86	1.56
Past-year workplace use	38.44	25.66	51.21	31.15	18.51	43.79	2.00	1.36	2.93	1.55	0.97	2.46
Non-safety-sensitive jobs												
No past-year use	4.15	2.87	5.42	4.27	2.97	5.57	1.00			1.00		
Past-year non-workplace use	4.25	2.23	6.27	4.19	2.20	6.18	1.02	0.58	1.81	0.98	0.54	1.77
Past-year workplace use	14.72	4.75	24.69	12.30	5.21	19.40	3.54	1.68	7.46	2.87	1.48	5.57

Abbreviations: *AR*, absolute risk; *LCL*, lower confidence limit; *RR*, relative risk; *UCL*, upper confidence limit

^a^For analyses of all respondents, adjusted for age, sex, province/territory, education, general health, alcohol use, cigarette smoking, average weekly work hours, usual work schedule, job permanency, job tenure, supervisory role, safety-sensitive work, usual contact with supervisor, job visibility, industry, workplace size, workplace smoking restrictions, workplace substance use policy, survey wave, and survey mode. For stratified analyses, models were adjusted for the same covariates, excluding safety-sensitive work

When stratified by safety-sensitive work, ARs of workplace injury among workers in safety-sensitive jobs were 20.14% (95%CI 16.22–24.06) for those reporting no past-year use, 23.30% (95%CI 17.80–28.80) for those reporting non-workplace use, and 31.15% (95%CI 18.51–43.79) among workers reporting workplace use. ARs of workplace injury for workers in non-safety-sensitive jobs were 4.27% (95%CI 2.97–5.57) for no past-year use, 4.19% (95%CI 2.20–6.18) for non-workplace use, and 12.30% (95%CI 5.21–19.40) for workplace use. When compared to no past-year use, non-workplace use was not statistically significantly associated with the risk of workplace injury among workers in safety-sensitive and non-safety-sensitive jobs. On the other hand, for workers in both safety-sensitive (RR 1.55, 95%CI 0.97–22.46) and non-safety-sensitive (RR 2.87, 95%CI 1.48–5.57) jobs who reported workplace use, the risk of experiencing a workplace injury was elevated compared to workers not using cannabis in the past year, though this finding was not statistically significant for workers in safety-sensitive jobs.

## Discussion

In this longitudinal study, we evaluated the relationship between past-year cannabis use and the risk of workplace injury, differentiating workers who used cannabis before and/or at work (workplace use) from those using outside of work only (non-workplace use). While no statistically elevated relationship existed between non-workplace use and workplace injury, workplace use was associated with an almost two-fold increase in the risk of workplace injury. This pattern of findings was seen among workers in both safety-sensitive and non-safety-sensitive jobs.

Study results bring greater clarity to the question of whether cannabis use increases the risk of experiencing a workplace injury, an issue that the conflicting findings of previous studies have hampered. Findings suggest that, when thinking about the potential occupational safety impacts of a worker’s cannabis use, it is important to consider when that use is taking place. More specifically, only use in close temporal proximity to work appears to be a risk factor for workplace injuries, not use away from work. Our findings support Frone’s conceptual model of worker substance use and workplace productivity (Frone, 2019). Our results are also consistent with at least one previous study of employed adolescents that found workplace substance use (alcohol and cannabis combined) was associated with greater odds of workplace injury, but not general substance use (Frone, 1998). Another study found workplace cannabis use to be associated with poor work performance, while no relationship was seen for after-work use (Bernerth & Walker, 2020).

Further, the findings of our study may also explain the source of inconsistencies in prior research on cannabis use and workplace injury. Whether or not cannabis use was associated with workplace injury in past research was likely a function of the proportion of the sample engaging in workplace use. A study including a small proportion of workers engaging in workplace use may have null findings, and a larger proportion may result in a significant positive association. Therefore, assessments of general cannabis use may not lead to appropriate conclusions. In our sample, ~ 18% of respondents reporting past-year cannabis use used before and/or at work. If we had only considered any past-year cannabis use, we would have found that cannabis use was only marginally associated with an increased risk of workplace injury compared to no use (RR 1.25, 95%CI 0.96–1.63; details upon request). In addition, we would have missed the critical contribution of the context of this use. This has implications for how studies examining this issue may inform future research and workplace policy. When using measures of general cannabis use, null results suggest cannabis is not a concern for workplace injuries, while a significant positive association suggests that *any* cannabis use is problematic and should be the focus of research and policy aimed at injury reduction. However, our results clearly demonstrate that by considering the temporal context of cannabis use, future research, workplace interventions, and workplace policies focusing on workplace injury mitigation should focus on cannabis use before or during work hours, which can result in cognitive and psychomotor impairment on the job.

Study findings also suggest that, irrespective of whether a worker’s job is safety-sensitive, only workplace cannabis use poses a risk to future workplace injury. Using relative effect measures, the risk associated with workplace use was larger among workers in non-safety-sensitive jobs (RR 2.87) than in safety-sensitive work (RR 1.55). However, this finding should be interpreted along with the absolute risks. The baseline risk (among unexposed) was considerably higher among those in safety-sensitive jobs (20.14%) compared to those in non-safety-sensitive jobs (4.27%). This likely contributed to the larger relative increase in risk among workers in non-safety-sensitive work. Furthermore, injuries incurred by those in safety-sensitive positions are more likely to be severe. Still, the increase in risk associated with workplace cannabis use among workers in non-safety-sensitive jobs should not be discounted, as it represents a preventable increase in risk.

Certainly, results from our study should be replicated in other samples. The findings also do not diminish employers’ legitimate concerns regarding workplace impairment. Nonetheless, zero-tolerance policies that prohibit cannabis use entirely, including use outside of work, may be overly broad and are incompatible with the results of this study. In an increasingly legalized environment, more nuanced approaches to workplace policies around cannabis use may be warranted, and could include employing minimum waiting periods after cannabis consumption when impairment is most likely present. For instance, it has been recommended that workers wait at least 6 to 12 h after inhalation and 8 to 12 h after ingesting cannabis before engaging in safety-sensitive tasks (MacCallum et al., 2022). The Occupational and Environmental Medical Association of Canada has more cautiously recommended waiting at least 24 h after consuming cannabis before engaging in safety-sensitive work (Occupational and Environmental Medical Association of Canada, 2018). Although more robust evidence on precise impairment windows is still required, using waiting periods in workplace policies derived from the best available evidence may be a reasonable approach and could include adding “safety cushions” to the length of the waiting period for safety-sensitive workplaces (Beckson et al., 2022).

### Strengths and limitations

This study addressed several limitations of previous research. First, the longitudinal study design ensured that cannabis exposure preceded the workplace injury outcome. Second, our contextual measure of cannabis use differentiated between non-workplace and workplace use. Third, the study sample was large and broadly represented workers from various occupations and industries, allowing for stratified analyses by the safety-sensitivity of the job. Finally, the findings were robust to adjustment for a wide range of potential confounders, and there was little item-level missing data.

The study also has limitations. We could not directly measure workplace impairment, nor could we account for the type of cannabis product used. In previous analyses (Carnide et al., 2021), we demonstrated that most workers reporting workplace use in our initial sample were using products with higher amounts of tetrahydrocannabinol (70.3%) or they did not know the amount (21.2%). However, due to questionnaire modifications, this information was not consistently recorded across survey cycles. Likewise, most of our sample used inhalation methods of consumption and we could not assess whether differences in risk exist between various forms of cannabis consumption. Our definition of use before work was limited to 2 h before work, which is likely insufficient to capture longer-lasting effects of ingestible methods. As such, study findings should not be interpreted as suggesting that 2 h be considered an appropriate cutoff beyond which using cannabis before work is safe. Furthermore, given that product type and method of consumption can influence the magnitude and duration of cannabis impairment, future research needs to assess the effects of different product formulations on risk of workplace injury.

The workplace injury outcome did not account for the severity or nature of the injury. Further, the lag between when cannabis use and workplace injury were measured may have resulted in some misclassification of exposure and attenuation of the findings. All data were based on self-report and social desirability bias may also have led to an underestimate of workplace cannabis use, and consequently, an underestimate of the association. Although we adjusted for several potential confounders, residual confounding is still possible, as we lacked information on other variables potentially associated with cannabis use and injury, such as co-occurring use of other substances, fatigue, and personality characteristics. Finally, the survey wave response rates were low (from 13.2% to 18.3%). However, the eligibility of those sampled but not contacted is unknown, making these rates conservative estimates of response. This large sample of Canadian workers is also similar in composition to the Canadian labour force, and those reporting cannabis use in this study exhibit a similar frequency of cannabis use, method of consumption, and age and sex demographics as seen in Canadian general population studies (Health Canada, 2020; Rotermann & Langlois, 2015).

## Conclusion

Workplace injuries pose a substantial burden on workers, employers, and society. Workplace cannabis use represents a preventable risk factor for workplace injuries. Although the prevalence of workplace cannabis use in the overall working population is relatively low, recent data have shown that, among workers who use cannabis, approximately one in four do so before or at work (Carnide et al., 2021; Health Canada, 2020). Furthermore, cannabis use among workers and working-aged adults is increasing (Carnide et al., 2022; Fischer et al., 2021; Frone, 2019). Over time, it is conceivable that workplace use may also increase. Although educational campaigns on cannabis impairment have primarily focused on preventing cannabis-impaired driving, our study findings demonstrate that workers are an important segment of the population who merit workplace-focused education on the risks of workplace cannabis use. Specific messaging around use before safety-sensitive work is also warranted, as some workers may be unsure of or perceive minimal safety risk of using cannabis before safety-sensitive work (Carnide et al., 2022). Workers in safety-sensitive jobs may also be more likely to engage in workplace use (Carnide et al., 2021).

Results of this novel study suggest workplace cannabis use, not use outside of work, is a risk factor for workplace injuries. Additional research examining the impact of specific cannabis product characteristics on the risk of workplace injury is warranted.

## Contributions to knowledge

What does this study add to existing knowledge?

- This study offers novel insights and greater clarity to the question of whether cannabis use increases the risk of experiencing a workplace injury, an issue that the conflicting findings of previous studies have hampered.
- Findings from this longitudinal study of Canadian workers clearly demonstrate that only cannabis use before and/or at work (workplace use) is a risk factor for workplace injuries, not use away from work (non-workplace use).
- This pattern of findings was seen among workers in both safety-sensitive and non-safety-sensitive jobs.

What are the key implications for public health interventions, practice, or policy?

- Workplace cannabis use represents a preventable risk factor for workplace injuries.
- While considerable efforts have been made to educate the public on cannabis-impaired driving, study findings underscore the need for worker-focused education on the risks of workplace cannabis use. Specific messaging around use before safety-sensitive work is important, given the potential catastrophic consequences of impairment in these roles.
- Findings suggest zero-tolerance workplace policies that prohibit cannabis use entirely, including use outside of work, may be overly broad. In an increasingly legalized environment, more nuanced approaches, such as employing minimum waiting periods after cannabis consumption, may be warranted.

### Supplementary Information

Below is the link to the electronic supplementary material.

Supplementary file1 (PDF 94 KB)

Supplementary file2 (PDF 304 KB)

## Data Availability

The data underlying this article may be shared on reasonable request by contacting the corresponding author.

## Code availability

The SAS and R codes used in the analyses are available on reasonable request by contacting the corresponding author.

## References

[CR1] Beckson M, Hagtvedt R, Els C (2022). Cannabis use before safety-sensitive work: What delay is prudent?. Neuroscience & Biobehavioral Review.

[CR2] Bernerth JB, Walker HJ (2020). Altered states or much to do about nothing? A Study of when cannabis is used in relation to the impact it has on performance. Group & Organization Management.

[CR3] Biasutti WR, Leffers KSH, Callaghan RC (2020). Systematic review of cannabis use and risk of occupational injury. Substance Use & Misuse.

[CR4] Broyd SJ, van Hell HH, Beale C, Yucel M, Solowij N (2016). Acute and chronic effects of cannabinoids on human cognition-A systematic review. [Review]. Biological Psychiatry.

[CR5] Carliner H, Brown QL, Sarvet AL, Hasin DS (2017). Cannabis use, attitudes, and legal status in the U.S.: A review. Preventive Medicine.

[CR6] Carnide N, Lee H, Frone MR, Furlan AD, Smith PM (2021). Patterns and correlates of workplace and non-workplace cannabis use among Canadian workers before the legalization of non-medical cannabis. Drug and Alcohol Dependence.

[CR7] Carnide N, Lee H, Landsman V, Frone MR, Furlan AD, Smith PM (2022). Cannabis use and workplace cannabis availability, perceptions and policies among Canadian workers: A comparison before and after the legalisation of non-medical cannabis. Occupational and Environmental Medicine.

[CR8] Els C, Straube S (2016). Marijuana and the workplace. The Canadian Journal of Addiction.

[CR9] Fischer B, Lee A, Robinson T, Hall W (2021). An overview of select cannabis use and supply indicators pre- and post-legalization in Canada. Substance Abuse: Treatment, Prevention, and Policy.

[CR10] Frone MR (1998). Predictors of work injuries among employed adolescents. Journal of Applied Psychology.

[CR11] Frone MR (2003). Predictors of overall and on-the-job substance use among young workers. Journal of Occupational Health Psychology.

[CR12] Frone MR (2006). Prevalence and distribution of illicit drug use in the workforce and in the workplace: Findings and implications from a U.S. national survey. Journal of Applied Psychology.

[CR13] Frone MR (2019). Employee psychoactive substance involvement: Historical context, key findings, and future directions. Annual Review of Organizational Psychology and Organizational Behavior.

[CR14] Frone MR, Trinidad JR (2012). Relation of supervisor social control to employee substance use: Considering the dimensionality of social control, temporal context of substance use, and substance legality. Journal of Studies on Alcohol and Drugs.

[CR15] Graubard BI, Korn EL (1999). Predictive margins with survey data. Biometrics.

[CR16] Health Canada. (2017). The Canadian Cannabis Survey – Methodological report. http://epe.lac-bac.gc.ca/100/200/301/pwgsc-tpsgc/por-ef/health/2017/102-16-e/report.html. Accessed 25 Aug 2021.

[CR17] Health Canada. (2020). Canadian Cannabis Survey 2020: Summary. https://www.canada.ca/en/health-canada/services/drugs-medication/cannabis/research-data/canadian-cannabis-survey-2020-summary.html#a2. Accessed 25 Aug 2021.

[CR18] Howard J, Osborne J (2020). Cannabis and work: Need for more research. American Journal of Industrial Medicine.

[CR19] Leung J, Chan G, Stjepanovic D, Chung JYC, Hall W, Hammond D (2022). Prevalence and self-reported reasons of cannabis use for medical purposes in USA and Canada. Psychopharmacology (Berl).

[CR20] Localio AR, Margolis DJ, Berlin JA (2007). Relative risks and confidence intervals were easily computed indirectly from multivariable logistic regression. Journal of Clinical Epidemiology.

[CR21] MacCallum CA, Lo LA, Pistawka CA, Christiansen A, Boivin M, Snider-Adler M (2022). A clinical framework for assessing cannabis-related impairment risk. Frontiers in Psychiatry.

[CR22] Myran DT, Staykov E, Cantor N, Taljaard M, Quach BI, Hawken S (2022). How has access to legal cannabis changed over time? An analysis of the cannabis retail market in Canada 2 years following the legalisation of recreational cannabis. Drug and Alcohol Review.

[CR23] Occupational and Environmental Medical Association of Canada. (2018). *Position statement on the implications of cannabis use for safety-sensitive work*. https://oemac.org/wp-content/uploads/2018/09/Position-Statement-on-the-Implications-of-cannabis-use.pdf. Accessed 1 Oct 2018.

[CR24] Rotermann M, Langlois K (2015). Prevalence and correlates of marijuana use in Canada, 2012. Health Reports.

[CR25] Rubin DB (1987). Multiple imputation for nonresponse in surveys.

[CR26] Smith PM, Saunders R, Lifshen M, Black O, Lay M, Breslin FC (2015). The development of a conceptual model and self-reported measure of occupational health and safety vulnerability. Accident Analysis & Prevention.

[CR27] Statistics Canada. (2016a). *Canadian community health survey (CCHS)*. http://www23.statcan.gc.ca/imdb/p2SV.pl?Function=getSurvey&Id=259374. Accessed 25 Aug 2021.

[CR28] Statistics Canada. (2016b). *Labour force survey questionnaire*. http://www23.statcan.gc.ca/imdb-bmdi/instrument/3701_Q1_V6-eng.htm. Accessed 25 Aug 2021.

[CR29] Statistics Canada. (2017a). *Canadian tobacco, alcohol and drugs survey (CTADS)*. Detailed information for 2017a. http://www23.statcan.gc.ca/imdb/p2SV.pl?Function=getSurvey&Id=333871. Accessed 25 Aug 2021.

[CR30] Statistics Canada. (2017b). *North American industry classification system (NAICS)*. Canada 2017b Version 3.0. http://www23.statcan.gc.ca/imdb/p3VD.pl?Function=getVD&TVD=1181553. Accessed 5 Sept 2019.

