# Supplementary material for: Workplace and non-workplace cannabis use and the risk of workplace injury: Findings from a longitudinal study of Canadian workers
Source: Can J Public Health. 2023 Jul 31;114(6):947–55. doi: 10.17269/s41997-023-00795-0 (PMC10661545; doi:10.17269/s41997-023-00795-0)
Supplement: Supplementary file 1 — Supplementary file1 (PDF 94 KB) [file 41997_2023_795_MOESM1_ESM.pdf]

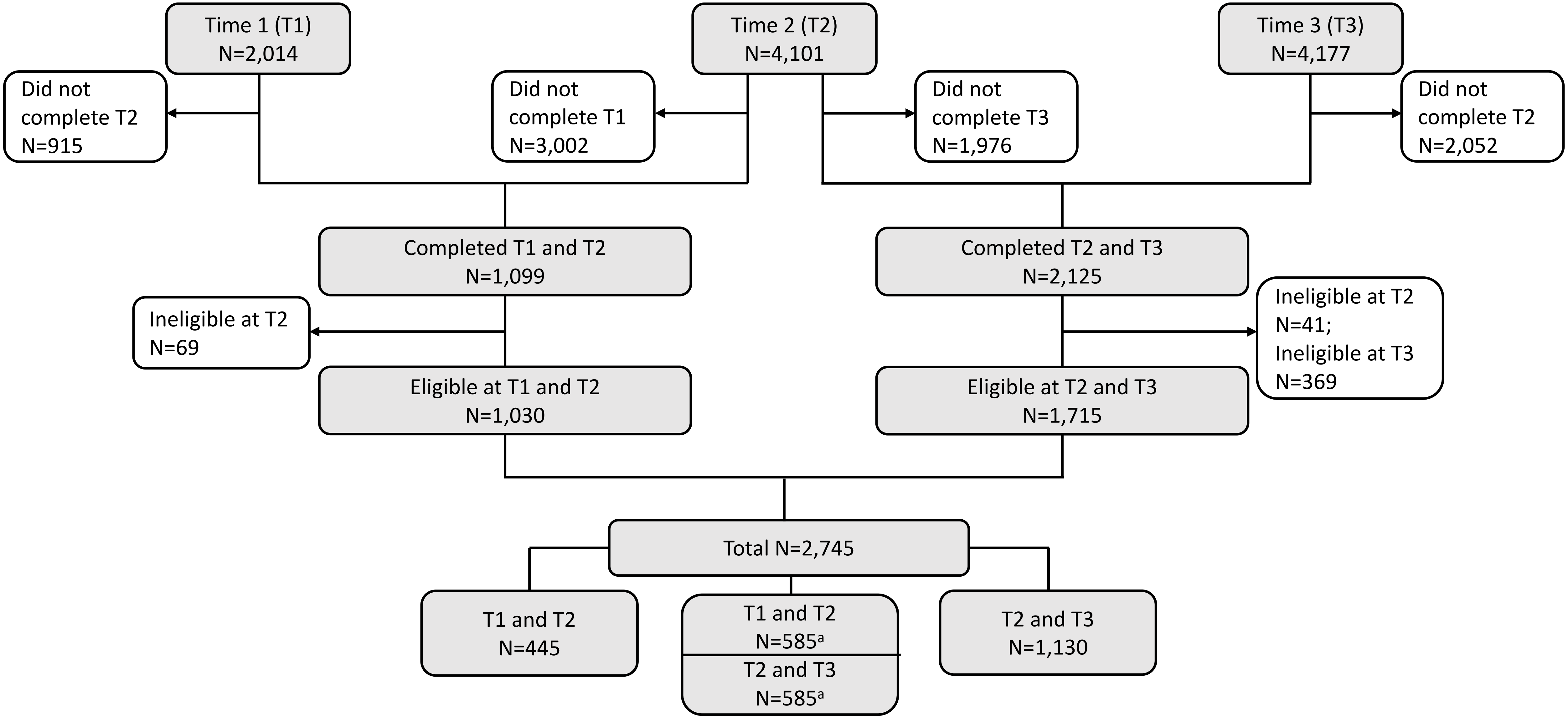

Figure S1 Flow of participants included in the final analysis examining workplace and non-workplace cannabis use and the risk of workplace injury

[a] A total of 585 participants participated in all three surveys. These individuals contributed two observations to the final analytic sample: T1 and T2 and T2 and T3.

Derivation of the analytic sample

The analysis is limited to workers participating in at least two adjacent surveys from the first three yearly waves of the study conducted from 2018 to 2020. A total of 2,014 individuals met eligibility and completed the survey at Time 1 [T1], 4,101 completed the survey at Time 2 [T2], and 4,177 completed the survey at Time 3 [T3] (Figure S1). Among the individuals participating in the T1 and/or T2 surveys, 915 participated at only T1 and 3,002 participated at only T2. This left 1,099 who participated at both T1 and T2, of which 69 were excluded as they no longer met study eligibility at T2. Therefore, a total of 1,030 individuals were eligible and participated at adjacent surveys T1 and T2.

Among the individuals participating in T2 and/or T3 surveys (Figure S1), 1,976 participated only at T2 and 2,052 participated only at T3, leaving 2,125 participants who completed both the T2 and T3 surveys. From this group, 410 were excluded due to ineligibility at T2 or T3, for a total of 1,715 individuals participating in adjacent surveys T2 and T3.

As shown at the bottom of Figure S1, the final analytic sample included 2,745 participants: 445 who only completed the T1 and T2 surveys, 1,130 who only completed the T2 and T3 surveys, and 585 who completed all three surveys. Note that the 585 participants who completed all three surveys appear twice in the final analytic sample, contributing one set of data from T1 and T2 and another set from T2 and T3.
