# Supplementary material for: Workplace and non-workplace cannabis use and the risk of workplace injury: Findings from a longitudinal study of Canadian workers
Source: Can J Public Health. 2023 Jul 31;114(6):947–55. doi: 10.17269/s41997-023-00795-0 (PMC10661545; doi:10.17269/s41997-023-00795-0)
Supplement: Supplementary file 2 — Supplementary file2 (PDF 304 KB) [file 41997_2023_795_MOESM2_ESM.pdf]

Supplementary Table S1. Personal and health-related characteristics of survey respondents, as observed in the sample (n=2,745[a]) and after multiple imputation (unweighted data)

	Observed Sample		Imputed Data[b]		
**Characteristics**	**N**	**%**	**%**	**95% LCL**	**95% UCL**
**Age in years, mean (SD)**	46.2	(11.1)			
**Sex**					
Male	1,605	58.5	58.5	56.6	60.3
Female	1,139	41.5	41.5	39.7	43.4
Missing	1	0.0			
**Region**					
British Columbia / Territories	328	12.0			
Alberta	345	12.6			
Saskatchewan / Manitoba	212	7.7			
Ontario	1,079	39.3			
Quebec	556	20.3			
Atlantic Region	225	8.2			
**Highest level of education**					
High school diploma or below	298	10.9	10.9	9.7	12.0
At least some post-secondary education	2,444	89.0	89.1	88.0	90.3
Missing	3	0.1			
**Self-rated general health**					
Good / Fair / Poor	1,200	43.7	43.8	41.9	45.6
Very good / Excellent	1,542	56.2	56.2	54.4	58.1
Missing	3	0.1			
**Past-year frequency of alcohol consumption**					
Never / Less than once a month	732	26.7	26.8	25.1	28.5
1 to 3 times a month	700	25.5	25.8	24.1	27.4
1 to 3 times a week	859	31.3	31.4	29.7	33.2
4 to 7 times per week	437	15.9	16.0	14.6	17.4
Missing	17	0.6			
**Current frequency of cigarette smoking**					
Not at all	2,349	85.6	85.6	84.3	86.9
Occasionally	135	4.9	5.0	4.2	5.8
Daily	257	9.4	9.4	8.3	10.5
Missing	4	0.2			
**Workplace injury in previous year**					
Yes	261	9.5	9.6	8.5	10.7
No	2,476	90.2	90.4	89.3	91.5
Missing	8	0.3			

Abbreviations: LCL, lower confidence limit; SD, standard deviation; UCL, upper confidence limit.
[a] Out of 2,745, a total of 585 respondents contribute two observations.
[b] No imputed information is provided when data are complete (no missing information). Applicable to age and region.

Supplementary Table S2. Work and workplace characteristics of survey respondents, as observed in the sample (n=2,745[a]) and after multiple imputation (unweighted data)

	Observed Sample		Imputed Data[b,c]		
**Characteristics**	**N**	**%**	**%**	**95% LCL**	**95% UCL**
**Usual hours worked per week, mean (SD)**	38.8	(7.5)			
**Usual work schedule**					
Regular day, evening or night shift	2,347	85.5	85.6	84.2	86.9
Non-regular shift (e.g., rotating, split, on call, irregular)	393	14.3	14.4	13.1	15.8
Missing	5	0.2			
**Has a permanent job**					
Yes	2,520	91.8	91.9	90.9	92.9
No	219	8.0	8.1	7.1	9.1
Missing	6	0.2			
**Job tenure (years), mean (SD)[d]**	11.3	(9.5)	11.3	(10.9,	11.6)
**Industry**					
Arts, retail trade, accommodations	235	8.6	8.8	7.7	9.8
Health care and social assistance	327	11.9	11.9	10.7	13.1
Education	449	16.4	16.4	.	.
Manufacturing, trade, transportation, warehousing	375	13.7	13.9	12.6	15.2
Primary industry (including construction)	277	10.1	10.1	9.0	11.3
Other (including information, technology, finance, administrative, professional, science, public administration)	1,067	38.9	39.0	37.1	40.8
Missing	15	0.6			
**Safety-sensitive job**					
Yes	1,015	37.0	37.0	35.2	38.9
No	1,726	62.9	63.0	61.1	64.8
Missing	4	0.2			
**Has a supervisory role**					
Yes	1,193	43.5	43.6	41.8	45.5
No	1,543	56.2	56.4	54.5	58.2
Missing	9	0.3			
**Frequent contact with supervisor**					
Agree / Strongly agree	1,588	57.9	57.9	56.1	59.8
Neither agree nor disagree / Disagree / Strongly disagree	1,152	42.0	42.1	40.2	43.9
Missing	5	0.2			

Characteristics	Observed Sample		Imputed Data[b,c]		
	N	%	%	95% LCL	95% UCL
**Frequency of performing job duties in front of / near people**					
Never / Occasionally	432	15.7	15.8	14.4	17.2
Often	478	17.4	17.4	16.0	18.8
Very often	1,833	66.8	66.8	65.0	68.5
Missing	2	0.1			
**Workplace size**					
5-19	414	15.1			
20 to 99	815	29.7			
100 to 499	731	26.6			
≥500	785	28.6			
**Workplace has a substance use policy**					
Yes	2,045	74.5	74.5	72.9	76.2
No	291	10.6	10.7	9.5	11.8
Don't know	406	14.8	14.8	13.5	16.2
Missing	3	0.1			
**Workplace smoking restrictions**					
Restricted completely	957	34.9	35.1	33.3	36.9
Allowed in designated areas / Restricted in certain places	1,721	62.7	63.0	61.2	64.8
Not restricted at all	50	1.8	1.9	1.4	2.5
Missing	17	0.6			

Abbreviations: LCL, lower confidence limit; SD, standard deviation; UCL, upper confidence limit.

[a] Out of 2,745, a total of 585 respondents contribute two observations.

[b] No imputed information is provided when data are complete (no missing information). Applicable to hours worked per week and workplace size.

[c] Confidence intervals are not provided when no data are imputed for a particular category. Applicable to industry.

[d] Data on job tenure missing for n=12 respondents.

Supplementary Table S3. Cannabis use status and workplace injury among survey respondents, as observed in the sample (n=2,745[a]) and after multiple imputation (unweighted data)

	Observed Sample		Imputed Data[b]		
**Characteristics**	**N**	**%**	**%**	**95% LCL**	**95% UCL**
**Cannabis use**					
All respondents					
No past-year use	1820	66.3	66.8	65.0	68.6
Past-year non-workplace use	749	27.3	27.3	.	.
Past-year workplace use	134	4.9	5.9	5.0	6.9
Missing	42	1.5			
Respondents in safety-sensitive jobs[c]					
No past-year use	676	66.6	66.9	64.0	69.8
Past-year non-workplace use	271	26.7	26.7	23.9	29.4
Past-year workplace use	61	6.0	6.5	4.9	8.0
Missing	7	0.7			
Respondents in non-safety-sensitive jobs[c]					
No past-year use	1141	66.1	66.7	64.5	69.0
Past-year non-workplace use	478	27.7	27.7	25.5	29.8
Past-year workplace use	73	4.2	5.6	4.5	6.8
Missing	34	2.0			
**Workplace injury**					
All respondents					
Yes	299	10.9	11.0	9.9	12.2
No	2438	88.8	89.0	87.8	90.1
Missing	8	0.3			
Respondents in safety-sensitive jobs[c]					
Yes	213	21.0	21.2	18.7	23.8
No	797	78.5	78.8	76.2	81.3
Missing	5	0.5			
Respondents in non-safety-sensitive jobs[c]					
Yes	85	4.9	5.0	4.0	6.1
No	1639	95.0	95.0	93.9	96.0
Missing	2	0.1			

Abbreviations: LCL, lower confidence limit; SD, standard deviation; UCL, upper confidence limit.
[a] Out of 2,745, a total of 585 respondents contribute two observations.
[b] Confidence intervals are not provided when no data are imputed for a particular category.
[c] Information on safety-sensitive jobs were missing for 4 participants. Therefore, totals by safety-sensitive work do not add up to 2,745.

Supplementary Table S4. Past-year cannabis use patterns among all workers reporting past-year use and according to workplace/non-workplace cannabis use in the past year (n=893) (unweighted data)

Characteristics[b]	Use in the past year (n=893)[a] n (%)	Past-year non-workplace use (n=749)[a] n (%)	Past-year workplace use (n=134)[a] n (%)	*P*[c]
**Frequency of overall cannabis use**				<0.0001
<1 day per month	370 (41.4)	348 (46.5)	15 (11.2)	
1 to 3 days per month	173 (19.4)	151 (20.2)	22 (16.4)	
1 to 4 days per week	168 (18.8)	134 (17.9)	32 (23.9)	
5 to 7 days per week	182 (20.4)	116 (15.5)	65 (48.5)	
**Frequency of cannabis use before/at work**				
Never	749 (83.9)	749 (100)	0 (0.0)	
<1 day per month	42 (4.7)	0 (0.0)	42 (31.3)	
1 to 3 days per month	26 (2.9)	0 (0.0)	26 (19.4)	
At least 1 day/week	66 (7.4)	0 (0.0)	66 (49.3)	
**Purpose for use**				<0.0001
Non-medical purposes	597 (66.9)	528 (70.5)	67 (50.0)	
Medical purposes	60 (6.7)	40 (5.3)	18 (13.4)	
Both non-medical and medical purposes	226 (25.3)	178 (23.8)	47 (35.1)	
**Primary method of consumption[b]**				0.0151
Smoking	574 (64.3)	485 (64.8)	88 (65.7)	
Vaping	128 (14.3)	106 (14.2)	21 (15.7)	
Edibles (food, drink)	118 (13.2)	107 (14.3)	10 (7.5)	
Oil or tincture	55 (6.2)	46 (6.1)	9 (6.7)	
Other (dabbing, topical, multiple methods)	9 (1.0)	4 (0.5)	5 (3.7)	

[a] Data on past-year cannabis use was missing for 32 respondents. Sample sizes for past-year workplace use and past-year non-workplace use also do not add up to 893, due to missing data for an additional 10 individuals who reported using cannabis in the past year, but did not provide data on workplace use.

[b] Values may not add up to totals due to missing responses.

[c] P-values correspond to the results of chi-square analyses comparing past-year workplace use with past-year non-workplace use.
